# Supplementary figures and images for: Herpes simplex virus 2 (HSV-2) evolves faster in cell culture than HSV-1 by generating greater genetic diversity
Source: PLoS Pathog. 2021 Aug 26;17(8):e1009541. doi: 10.1371/journal.ppat.1009541 (PMC8389525; doi:10.1371/journal.ppat.1009541)

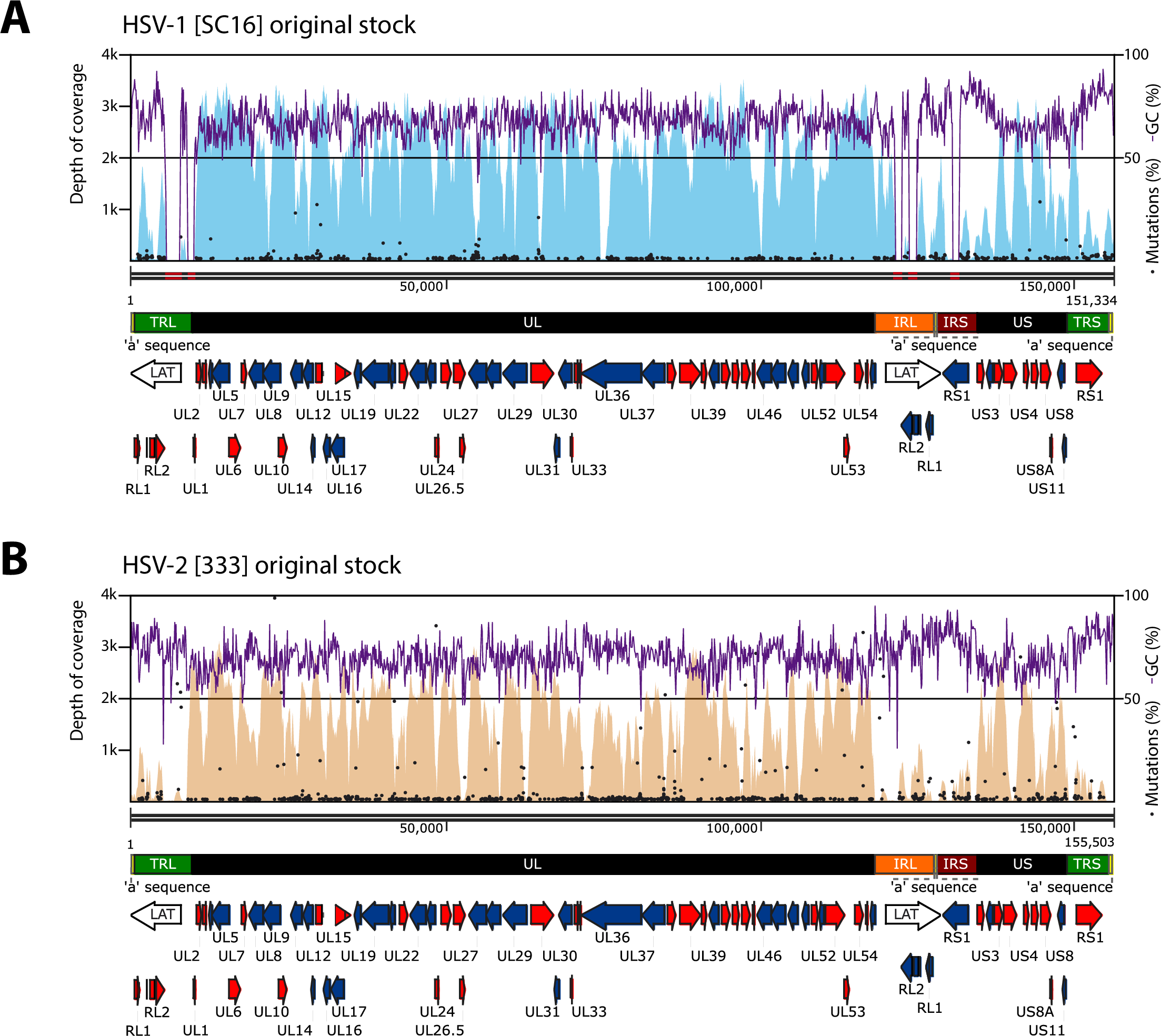

Supplement: S1 Fig — Schematic of the HSV-1 strain SC16 (A) and HSV-2 strain 333 (B) sequenced genomes from original stocks. Each CDS is presented in forward (red) or reverse (blue) orientation. Detected MVs (Sheets C and D in S1 Table) are mapped as black (not de novo) or red (de novo) dots across the genome, according to their location (x-axis) and frequency (y-axis). GC% plots (purple lines) and coverage plots from data alignments (blue/orange profiles) have also been mapped across each genome. (TIF) [file ppat.1009541.s001.tif]

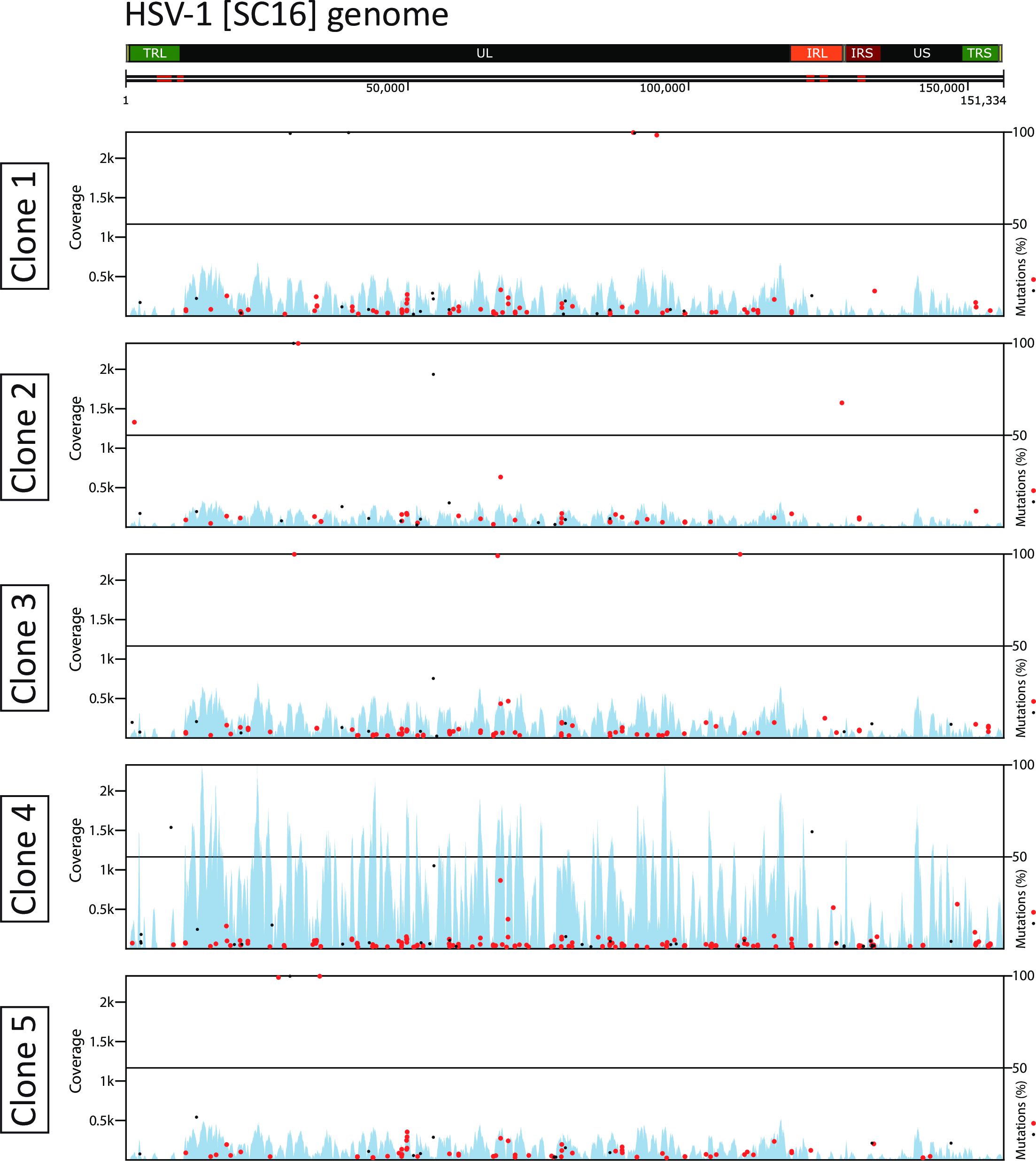

Supplement: S2 Fig — Coverage plots from data alignments are represented in blue, for each individual case. Detected MVs (Sheet C in S1 Table) are mapped as black (not de novo) or red (de novo) dots across the genome, according to their location (x-axis) and frequency (y-axis). MVs were considered as de novo when these were not previously found in the original stock (see Material and Methods for details). (TIF) [file ppat.1009541.s002.tif]

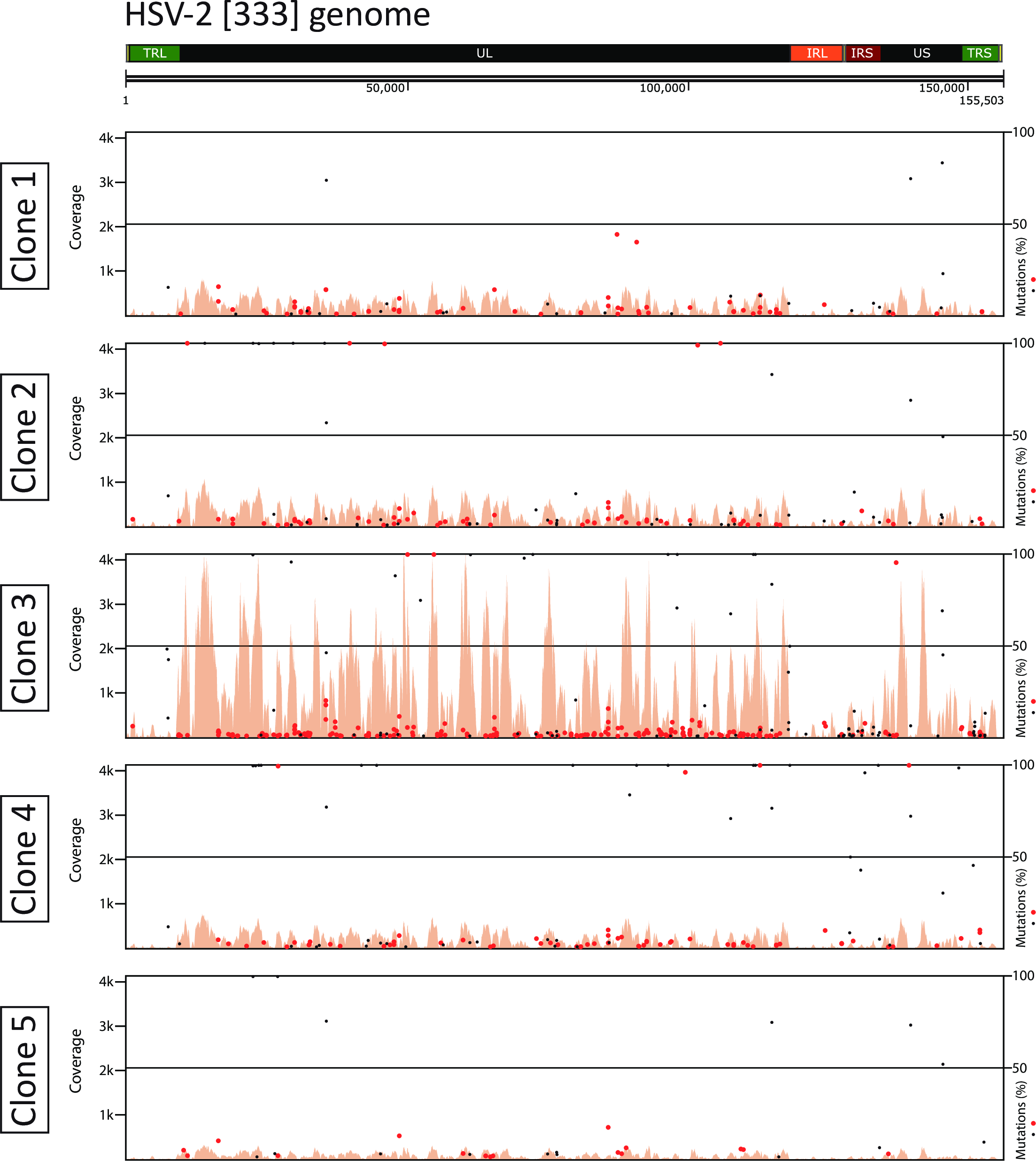

Supplement: S3 Fig — Coverage plots from data alignments are represented in orange, for each individual case. Detected MVs (Sheet D in S1 Table) are mapped as black (not de novo) or red (de novo) dots across the genome, according to their location (x-axis) and frequency (y-axis). MVs were considered as de novo when these were not previously found in the original stock. (TIF) [file ppat.1009541.s003.tif]

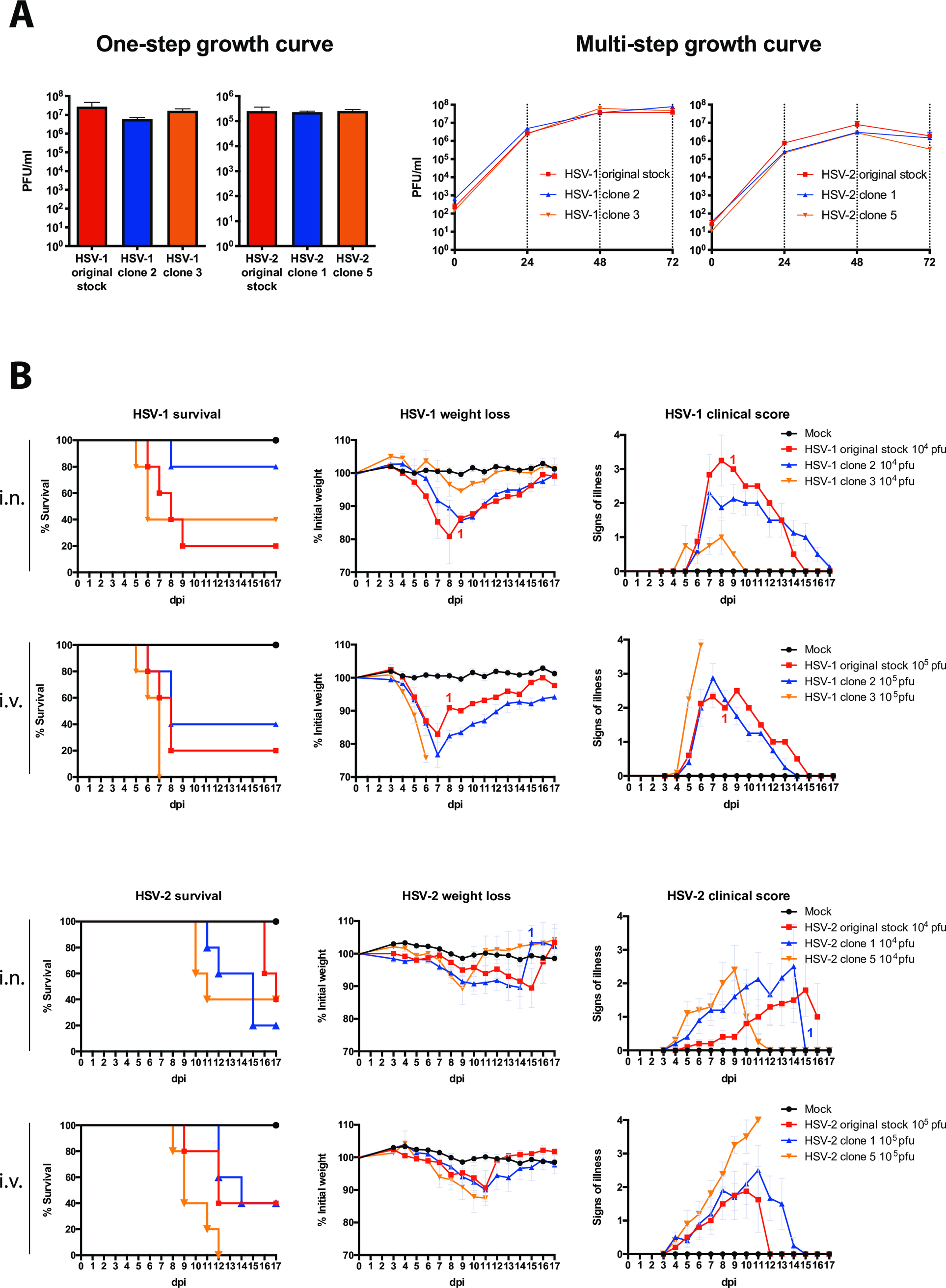

Supplement: S4 Fig — (A) Vero cells were infected with the indicated viruses at high MOI (5 PFU/cell) for one-step growth curves, and at low MOI (0.01 PFU/cell) for multi-step growth curves. Virus titers from fractions containing cell-associated virus were determined by plaque assay at 24 hpi in the one-step curves, and at the indicated times in the multi-step curves. Graphs display means and SD from two independent experiments performed in triplicate. (B) Female BALB/c mice (n = 5) were infected with the indicated virus and dose, by intranasal (i.n.) or intravaginal (i.v.) inoculations. Mice were monitored daily for survival, body weight, and signs of illness. Weight data are expressed as the mean +/- SEM of the five animal weights compared to their original weight on the day of inoculation. Signs of illness, as a score ranged from 1 to 4, is also expressed as the mean +/- SEM of the five animals. A colored “1” indicates thereafter only one animal remained in that group. Statistical analysis was performed for bodyweight data, using multiple t-tests with Sidak-Bonferroni correction (p < 0.05). (TIF) [file ppat.1009541.s004.tif]

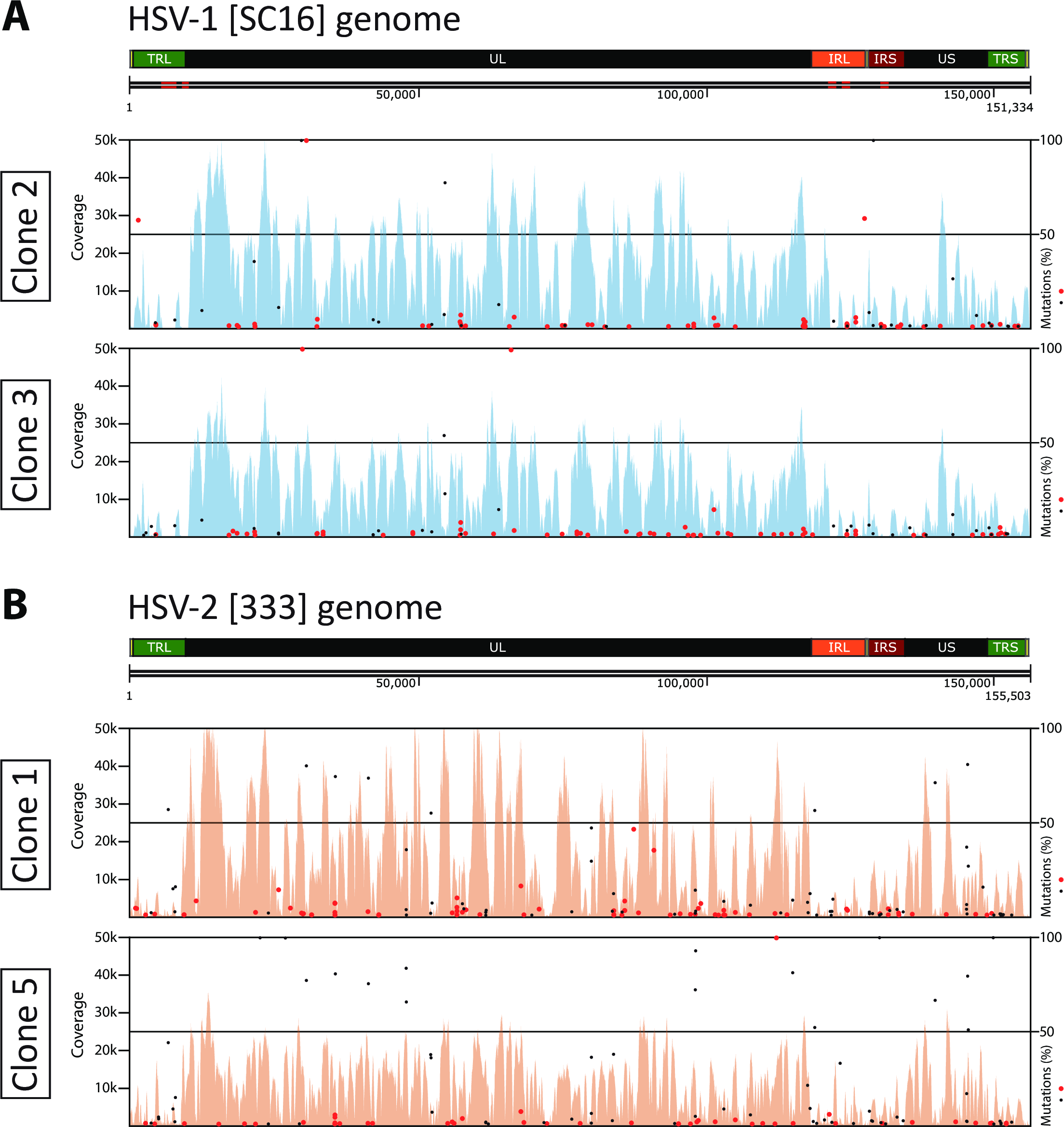

Supplement: S5 Fig — Variant analysis of HSV-1 plaque-purified clones 2 and 3 (A) and HSV-2 clones 1 and 5 (B) from high-depth sequencing data. Coverage plots from alignments are represented in blue or orange, for each case. Detected MVs (Sheets E and F in S1 Table) are mapped as black (not de novo) or red (de novo) dots across the genome, according to their location (x-axis) and frequency (y-axis). MVs were considered as de novo when these were not previously found in the corresponding original stock. (TIF) [file ppat.1009541.s005.tif]

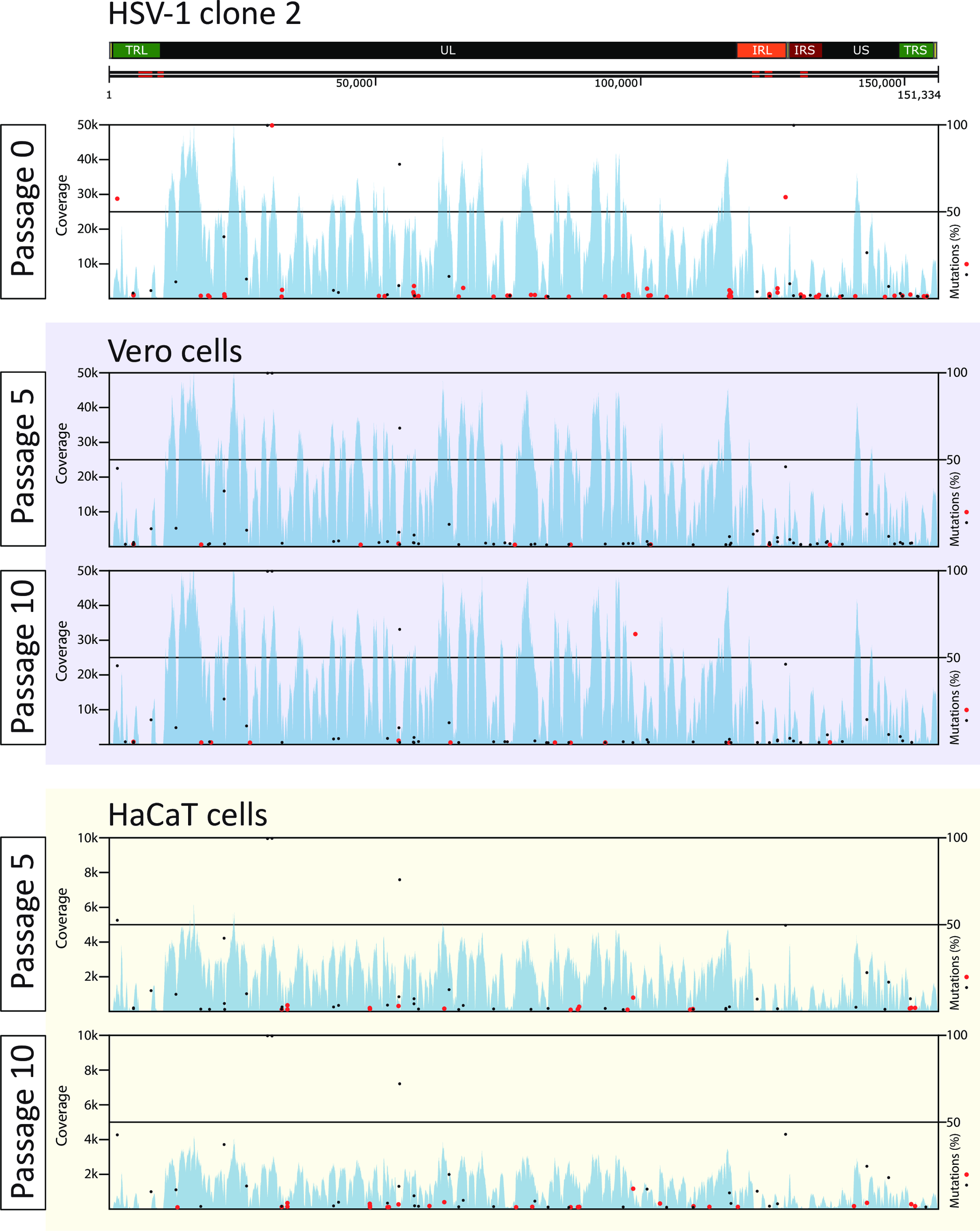

Supplement: S6 Fig — Coverage plots from high-depth sequencing data alignments are represented in blue. Detected MVs (Sheets G and H in S1 Table) are mapped as black (not de novo) or red (de novo) dots across the genome, according to their location (x-axis) and frequency (y-axis). Mutations from passage 0 were considered as de novo when these were not previously found in the original stock, whereas those from passage 5 and 10, regarding passage 0. (TIF) [file ppat.1009541.s006.tif]

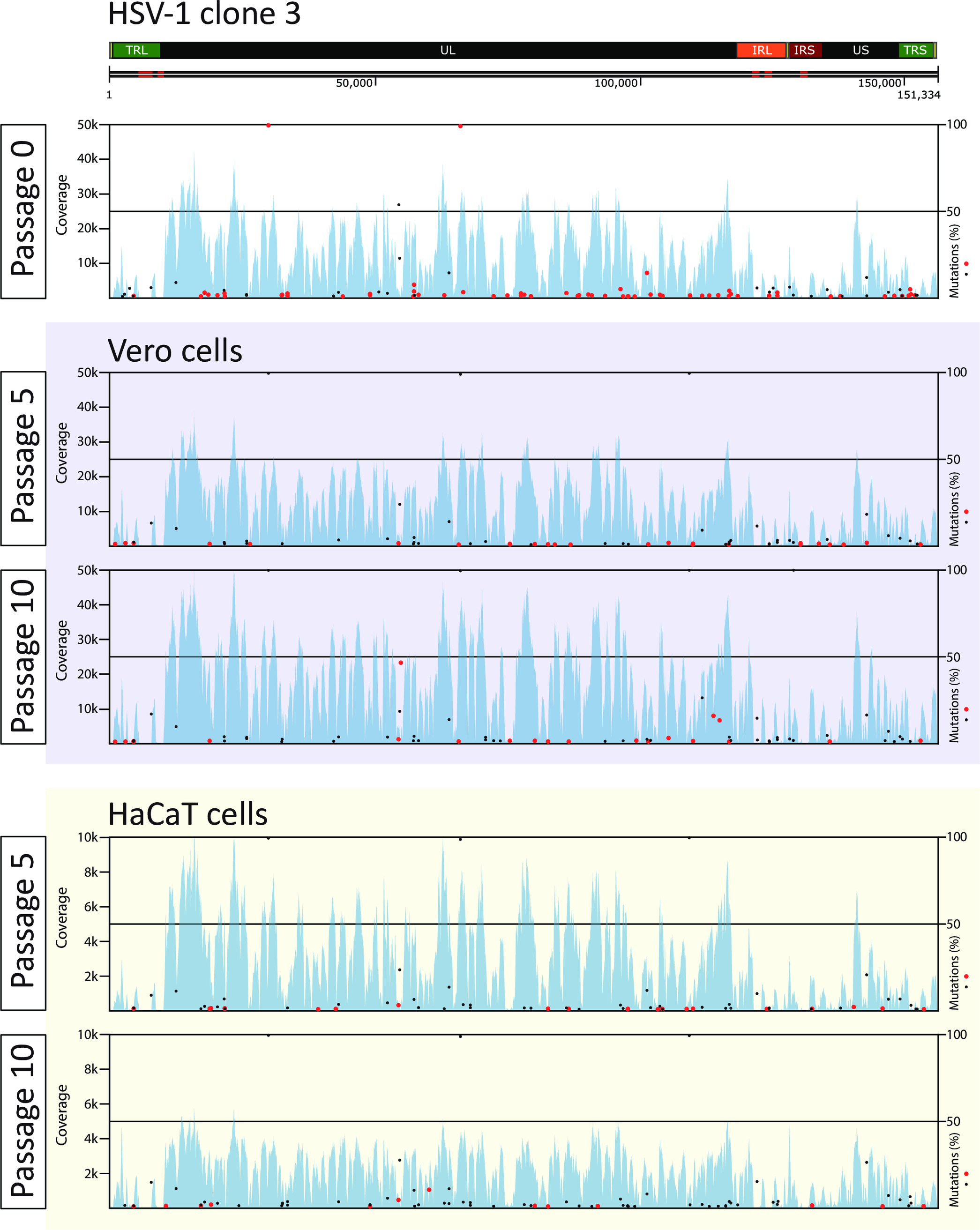

Supplement: S7 Fig — Coverage plots from high-depth sequencing data alignments are represented in blue. Detected MVs (Sheets I and J in S1 Table) are mapped as black (not de novo) or red (de novo) dots across the genome, according to their location (x-axis) and frequency (y-axis). Mutations from passage 0 were considered as de novo when these were not previously found in the original stock, whereas those from passage 5 and 10, regarding passage 0. (TIF) [file ppat.1009541.s007.tif]

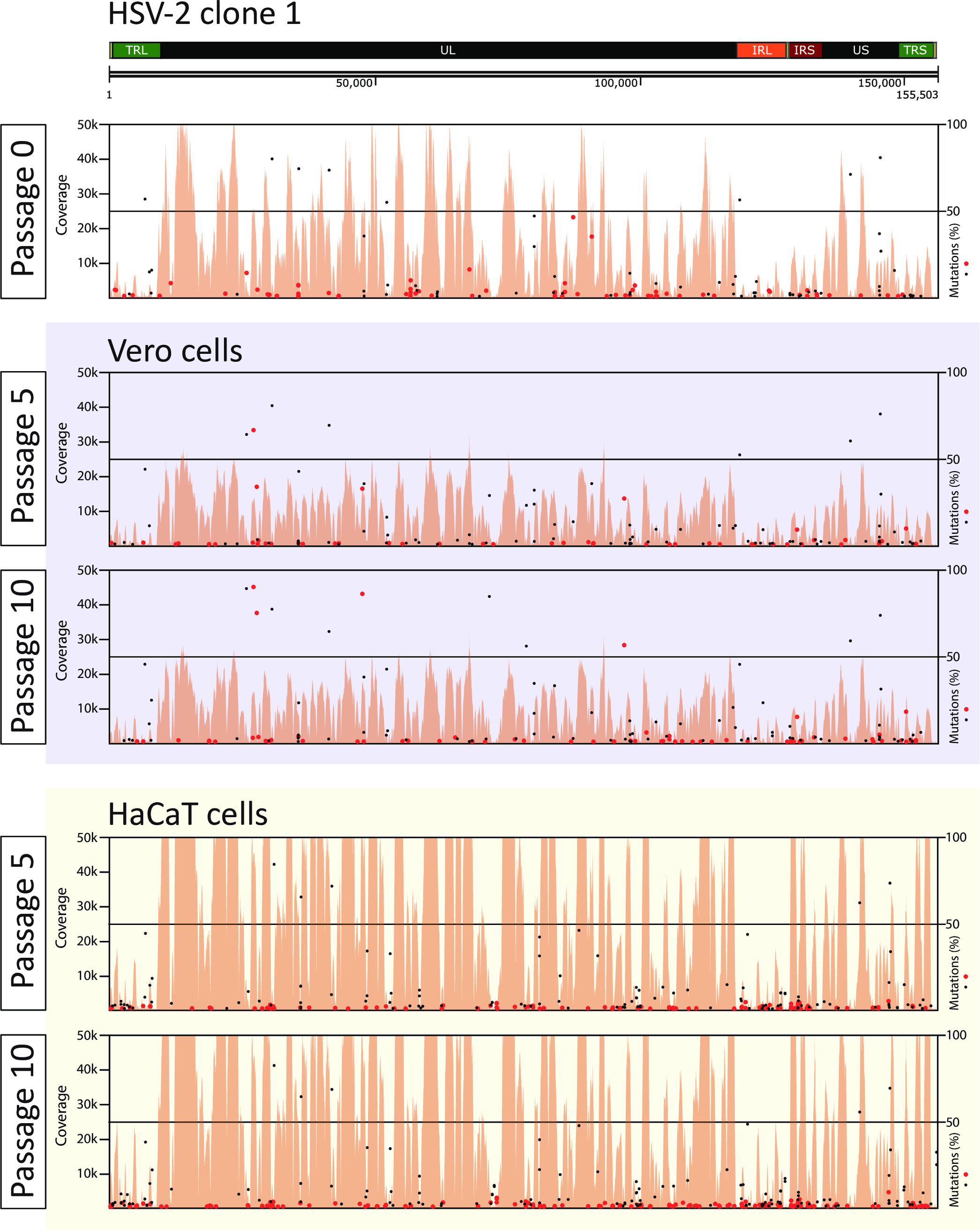

Supplement: S8 Fig — Coverage plots from high-depth sequencing data alignments are represented in orange. Detected MVs (Sheets K and L in S1 Table) are mapped as black (not de novo) or red (de novo) dots across the genome, according to their location (x-axis) and frequency (y-axis). Mutations from passage 0 were considered as de novo when these were not previously found in the original stock, whereas those from passage 5 and 10, regarding passage 0. (TIF) [file ppat.1009541.s008.tif]

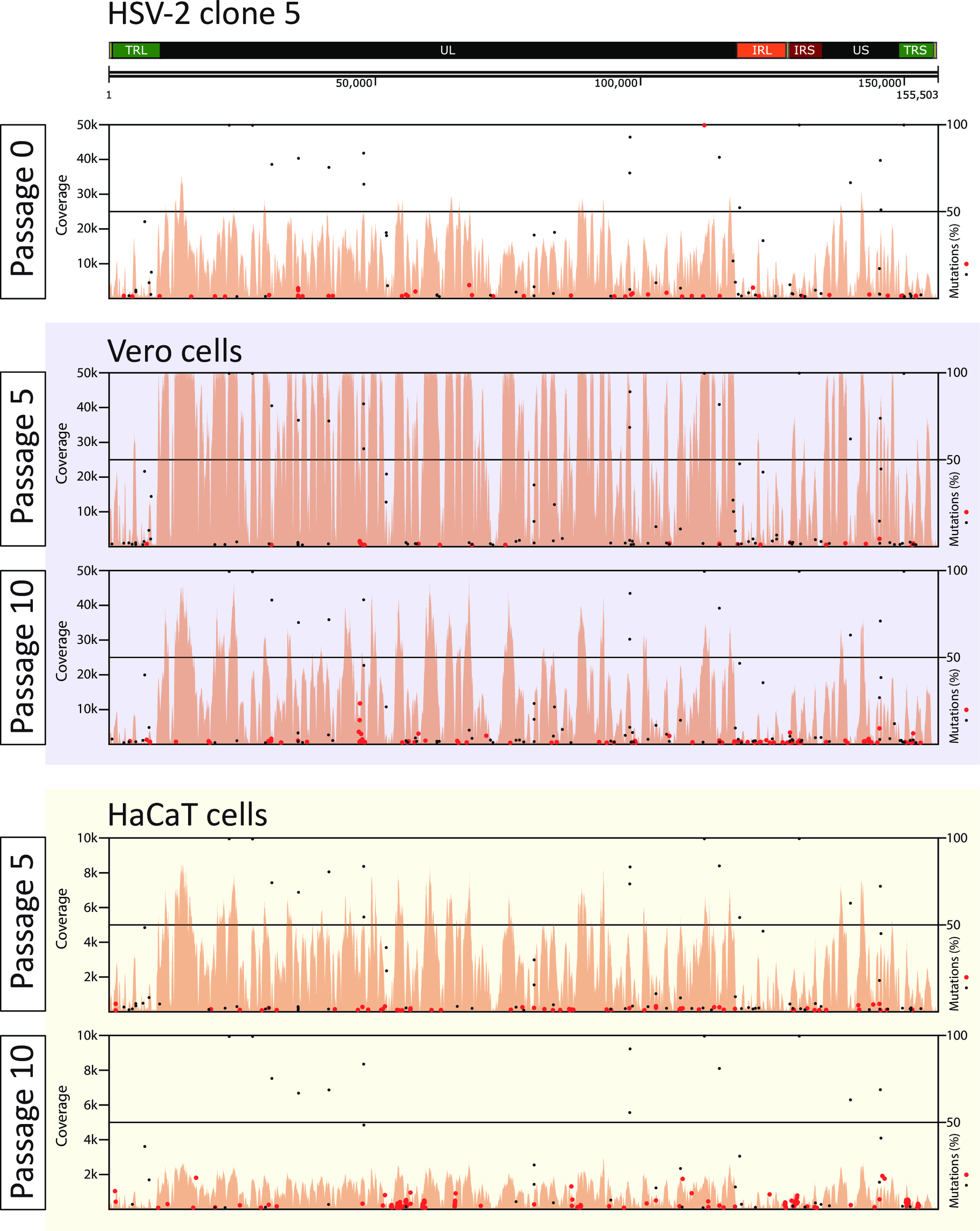

Supplement: S9 Fig — Coverage plots from high-depth sequencing data alignments are represented in orange. Detected MVs (Sheets M and N in S1 Table) are mapped as black (not de novo) or red (de novo) dots across the genome, according to their location (x-axis) and frequency (y-axis). Mutations from passage 0 were considered as de novo when these were not previously found in the original stock, whereas those from passage 5 and 10, regarding passage 0. (TIF) [file ppat.1009541.s009.tif]

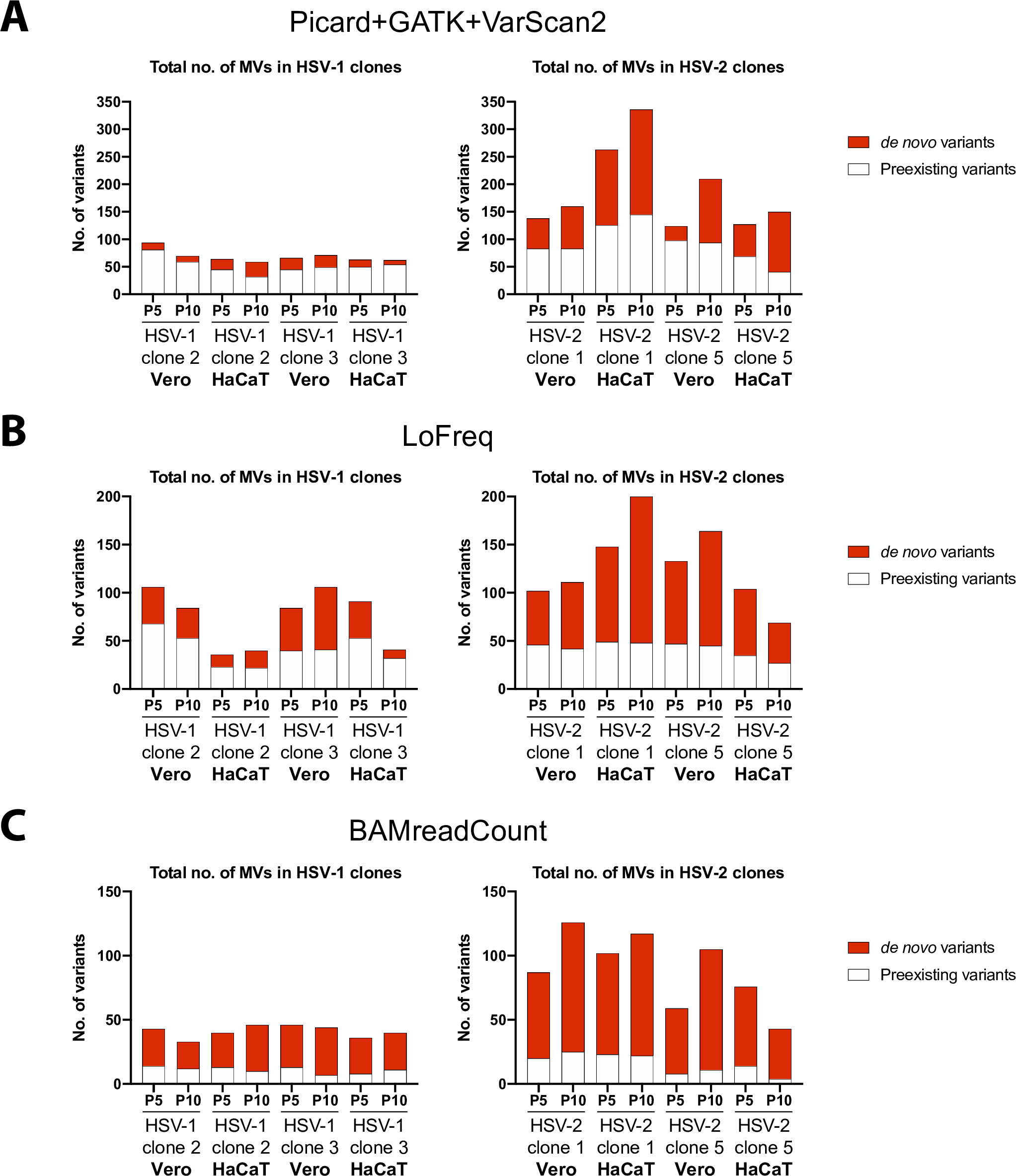

Supplement: S10 Fig — Total number of MVs are plotted according to variant analysis data (Sheets O and P in S1 Table) performed with Picard, GATK and VarScan2 (A), LoFreq (B), and BAMreadCount software (C). (TIF) [file ppat.1009541.s010.tif]

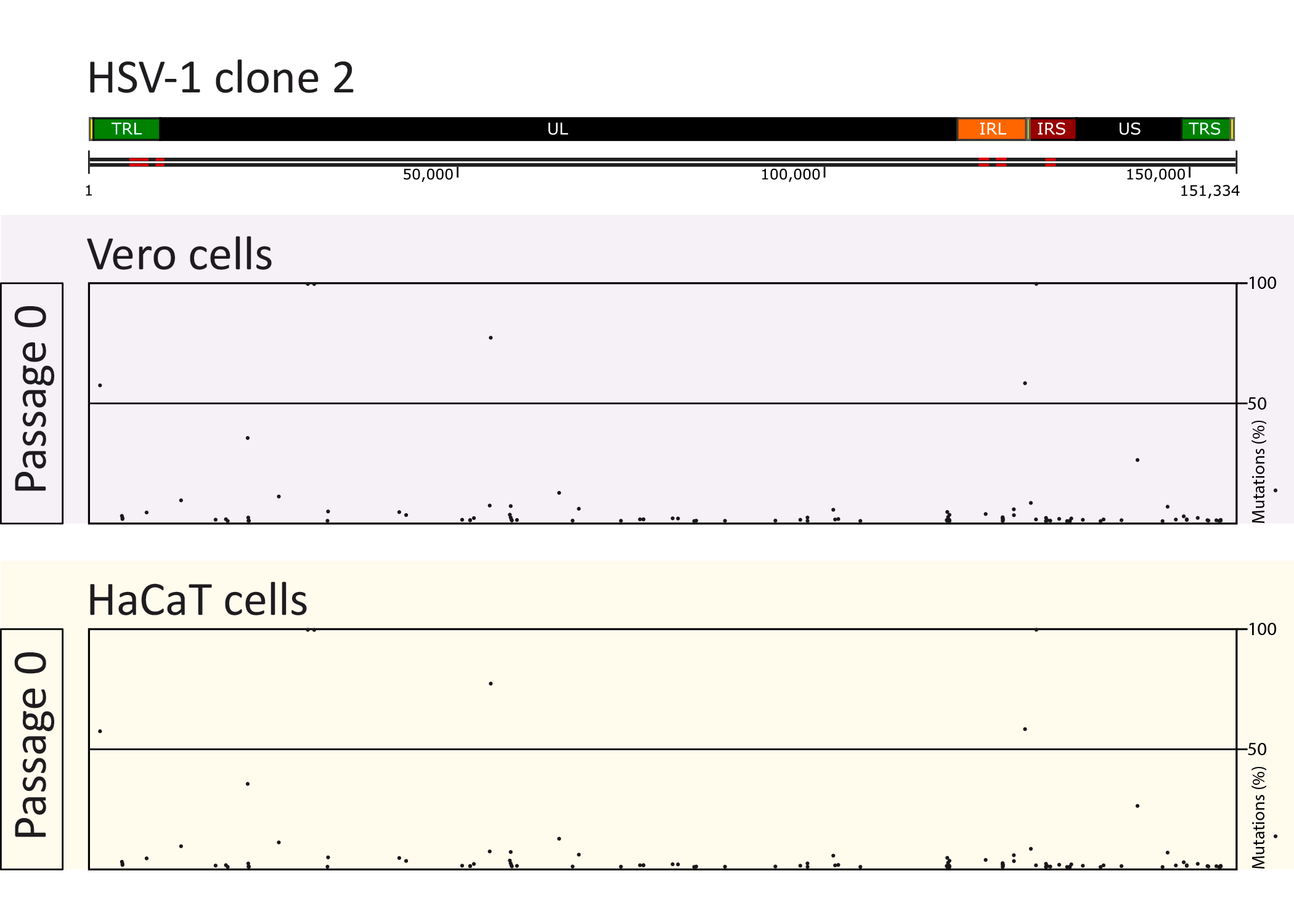

Supplement: S1 Animation — See S6 Fig for additional details. (GIF) [file ppat.1009541.s011.gif]

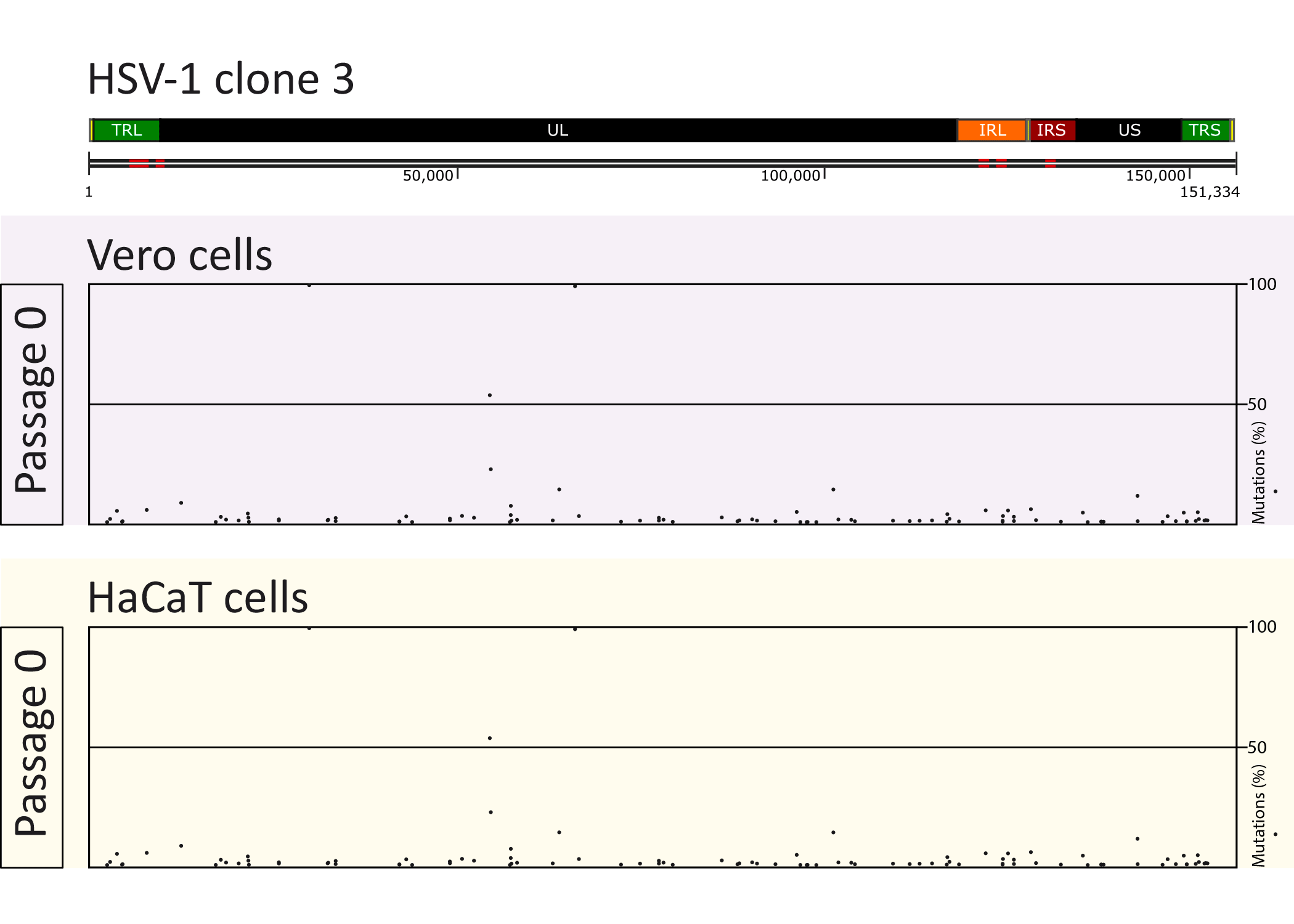

Supplement: S2 Animation — See S7 Fig for additional details. (GIF) [file ppat.1009541.s012.gif]

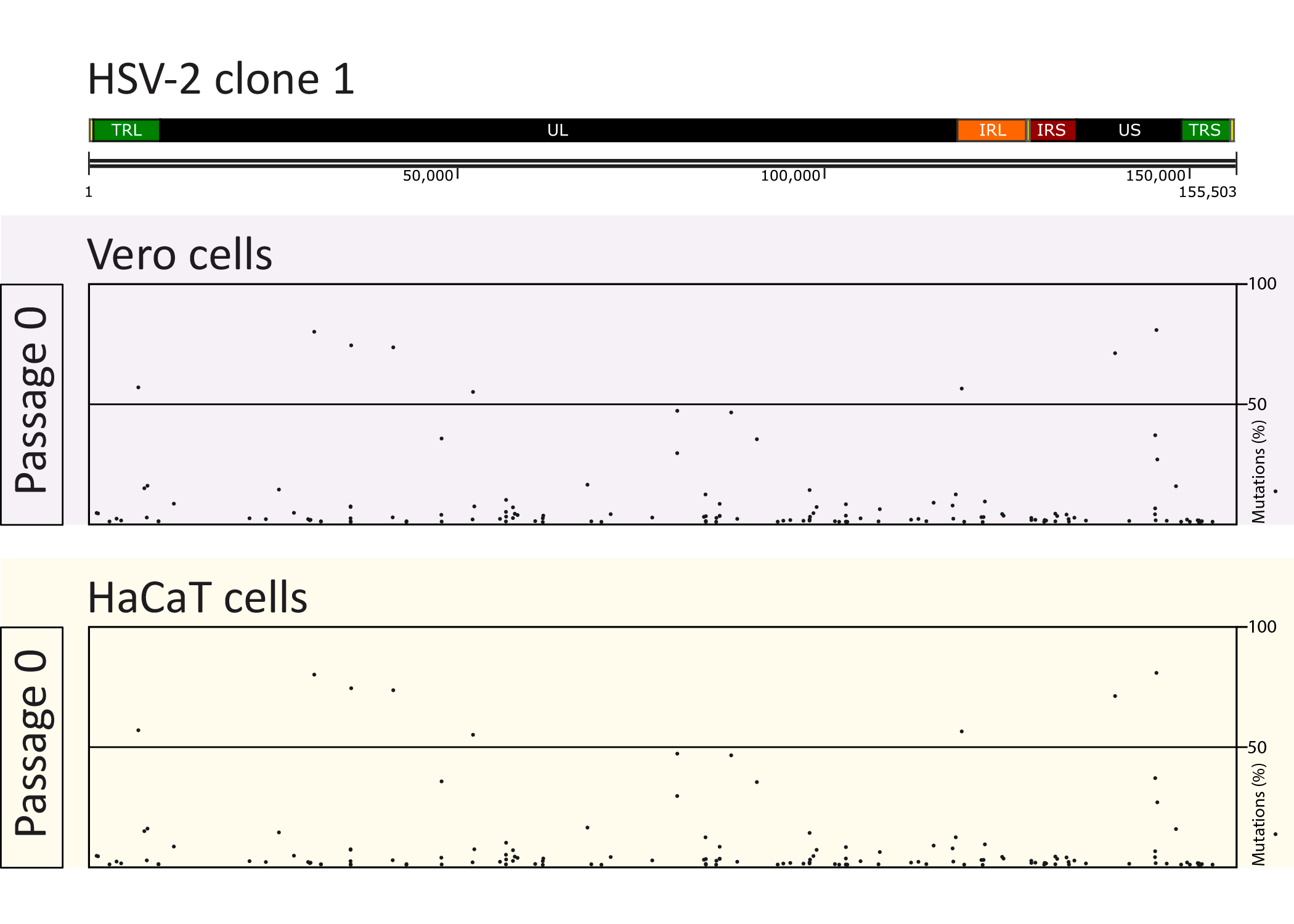

Supplement: S3 Animation — See S8 Fig for additional details. (GIF) [file ppat.1009541.s013.gif]

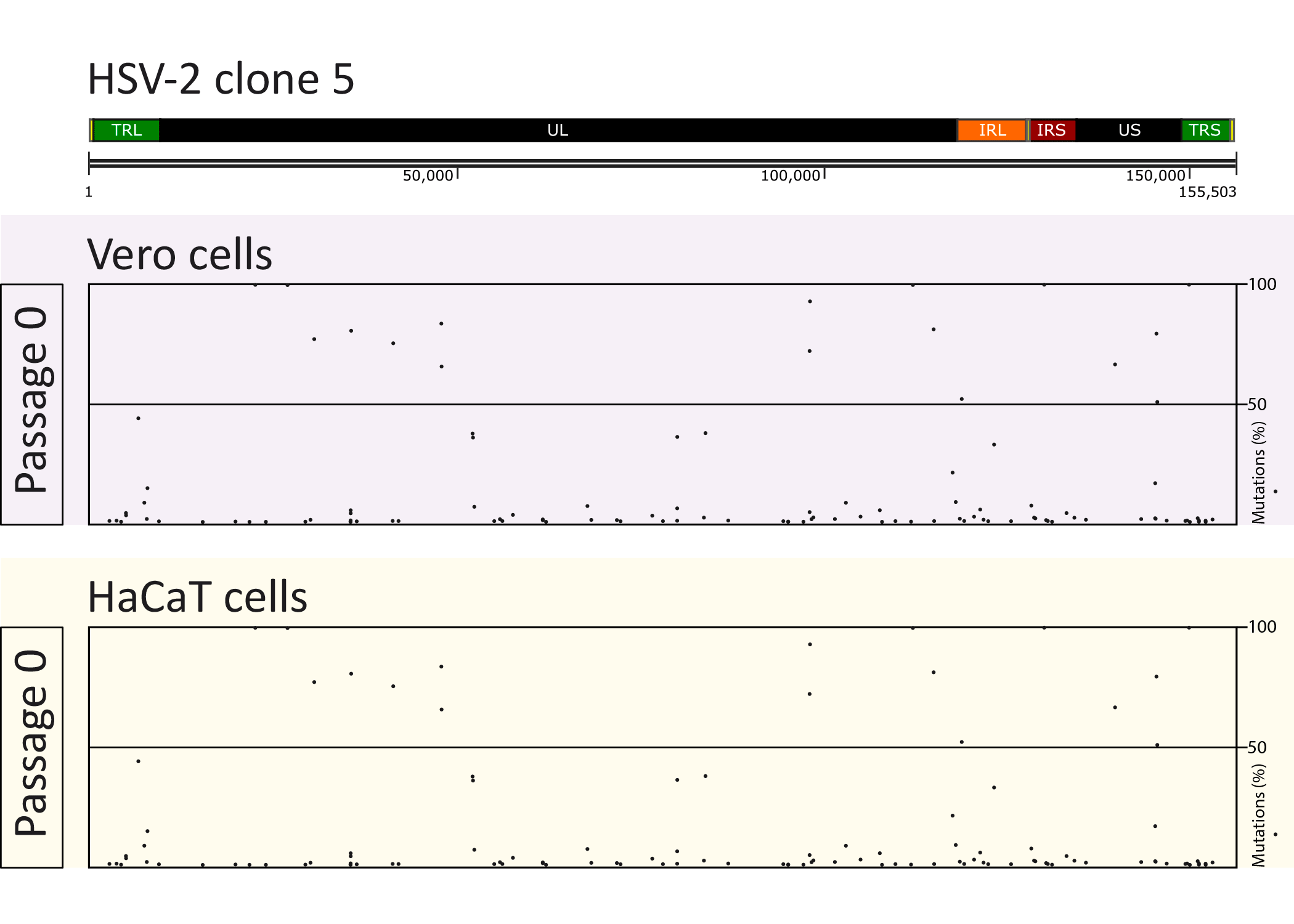

Supplement: S4 Animation — See S9 Fig for additional details. (GIF) [file ppat.1009541.s014.gif]
