# Supplementary material for: Herpes simplex virus 2 (HSV-2) evolves faster in cell culture than HSV-1 by generating greater genetic diversity
Source: PLoS Pathog. 2021 Aug 26;17(8):e1009541. doi: 10.1371/journal.ppat.1009541 (PMC8389525; doi:10.1371/journal.ppat.1009541)
Supplement: Attachment — Submitted filename: 0.Reviewers_ comments PPATHOGENS-D-21-00730_final.pdf [file ppat.1009541.s017.pdf]

PLOS Pathogens: Decision on your submission PPATHOGENS-D-21-00730 - [EMID:cbc3432658e7f223]

Dear Dr. Alcami,

We received back comments from the three reviewers who agreed to evaluate your manuscript. I do want to apologize for the extended time required to get the decision back to you as it was difficult to secure reviewers. The opinions of the reviewers were supportive and they all noted the value of the work. That being said, it appears that there were some significant concerns raised by the reviewers that would need to be addressed in order to secure an acceptance for publication in PLoS Pathogens. I would consider revisiting a revised version of the manuscript with the concerns of the reviewers addressed. Their specific concerns are listed below.

Cheers,

Eain Murphy.

Reviewer's Responses to Questions

Part I - Summary

Please use this section to discuss strengths/weaknesses of study, novelty/significance, general execution and scholarship.

Reviewer #1: HSV-1 and HSV-2 infections are prevalent and contribute to clinical pathology in patients worldwide. Recent studies done using high-throughput sequencing has revealed that these viruses are heterogeneous and rapidly evolve under a variety of selection pressures, including, but not limited to cell type, and understanding both the heterogeneity and the selection pressures that induce these genetic drifts is critical to understanding how these viruses could become resistant to antivirals, among other things. In this current manuscript, the authors performed standard deep sequencing and ultra deep sequencing on two commonly used viral stocks in HSV-1 and HSV-2 research labs and quantified the variants present in these stocks. Following plaque purification of clones from each stock, the authors reinvestigated variants in the clones after confirming that the virulence and in vivo phenotypes of the plaque purified clones was comparable to the original stocks. Importantly, they showed through deep sequencing of these passaged stocks that the earlier passages were more homogenous, and that with increasing passages the number of minor variants increased, and this was cell type dependent and finally HSV-2 variants were much more frequent. Overall, the

manuscript is well done and the data is convincing. The authors show clearly that even after relatively low passages of viral stocks, that genetic variants rapidly appear, a finding relevant to many researchers that utilize passaged HSV-1 and HSV-2 stocks in research today.

We appreciate the reviewer's comment.

Reviewer #2: In this article, "HSV-2 evolves faster in cell culture than HSV-1 by generating greater genetic diversity" by Lopez-Munoz, et al, the authors investigated the genetic variations in HSV-1 and HSV-2 in two cell types (Vero and HaCaT cells) over time by serial passage. The have compared deep and ultradeep sequencing of these passages over time to reveal genetic variation, including minor variants (MVs), SNPs, and InDels. Not surprisingly, the depth of sequencing increases the ability to detect genetic diversity present in low frequencies. While the sequencing data and analyses appear adequate, what is lacking is a connection to why this is important to viral fitness. While the authors performed growth curves, and one, very small in vivo experiment in mice, which are buried in the supplemental data, these experiments are just scratching the surface. As is, these data provide the foundation, but the subsequent analyses on the impact of viral infection, pathogenesis and spread are lacking. This is also important because it is not clear, at this point, whether this is simply a tissue culture phenomenon. If this data were from clinical samples over time, maybe this dataset would be ok, but it seems unsurprising that serial passage of any virus in tissue culture will result in genetic variations, if only to become more adapted to the tissue culture environment. In summary, while the sequencing data and analyses are adequate, this story is incomplete without accompanying data on how such diversity impacts the virus and/or virus-host relationship. Specifics are provided below.

We thank the reviewer for their observations. Despite being expectable, the fact that the depth of sequencing increases the ability to detect genetic diversity present in low frequencies, has not been previously assessed when characterizing genetic diversity of human herpesviruses. Most of previous studies reported an average coverage of 1000-3000x when performing analysis of minor variants [23, 30, 32, 34 – 41, 44]. These studies, among many others, did not perform a comparative and comprehensive analysis of the same sequenced sample(s) at different standard and ultra-deep sequencing. We provide the first experimental evidence of how ultra-deep sequencing can be used to reveal the very-low genetic diversity contained into a viral population. We believe that this is of high relevance and interest for the field.
There is increasing evidence showing the critical relevance of performing MV analysis to better understand viral fitness differences, where a single non-synonymous SNP can dramatically alter the viral phenotype [44, 50-52]. Here we have comprehensively characterized how HSV-1 and HSV-2 differentially evolve in

vitro, under equal, properly controlled conditions. We performed in vitro and in vivo studies of plaque-purified viral clones to corroborate that their biology was comparable to their original stocks after the reduction of genetic diversity (S4 Fig). We observed no differences in their viral phenotypes. The purpose of these studies was to establish a confident base line prior to assess the generation of genetic diversity in cell culture.

As commented in the introduction (lines 142-155), there has been increasing evidence reported of high intrahost genetic diversity from clinical isolates of HCMV [35-40], HSV-1 [32, 41] and HSV-2 [33]. This evidences that herpesviruses can generate genetic diversity in a few rounds of replication after reactivation from latency (from very few viral particles). Plaque-purified clones (this study) and mixed population of HSV-1 [44] are significantly less diverse than HSV-2 (this study), in cell culture. These observations support the idea that herpesviruses can differentially generate genetic diversity due to their intrinsic biology, rather than being beholding just a tissue culture phenomenon.

We believe that the demonstration of a constant generation of genetic variability, greater for HSV-2, is of high relevance. This means that these viruses have a better capacity to adapt to new environmental conditions than expected. Future studies will indeed be of high interest and will examine how this differential generation of genetic variably might be impacting the viral fitness in animal models. We believe that the impact of this variability to viral fitness in other, more relevant settings, such as the capacity of the virus to evade immune responses and contribute to pathogenesis in animal models, will be the subject of future studies driven in part by our observations.

Reviewer #3: López-Muñoz et al have performed a comparative study that aims to measure the rates at which HSV-1 and HSV-2 generate sequence diversity in vitro. Whether intentional or not, this study provocatively implies that HSV-1 and HSV-2 are capable of generating RNA virus-levels of diversity, a claim that has also been made for other herpesviruses (chiefly HCMV) and has also been subject to counter studies that refute these claims, most of which are due to technological/methodological artefacts. Significant care is therefore required both in the design and execution of experiments, but also in the interpretation and presentation of the data. The authors generally perform well in this regard but I do still have a few significant concerns that are detailed below and that I hope they are willing and able to address.

We also thank the reviewer for their comments. This study supports the idea that herpesviruses can differently change and adapt as dynamic populations, being less stable than previously assumed. This might reminisce to the quasispecies phenomenon traditionally associated with RNA viruses, but we do agree with

the fact that this would be a questionable claim, which is under discussion in the field and requires further investigation. We have addressed the rest of the concerns below.

Part II – Major Issues: Key Experiments Required for Acceptance
Please use this section to detail the key new experiments or modifications of existing experiments that should be absolutely required to validate study conclusions.

Generally, there should be no more than 3 such required experiments or major modifications for a "Major Revision" recommendation. If more than 3 experiments are necessary to validate the study conclusions, then you are encouraged to recommend "Reject".

Reviewer #1: NA

Reviewer #2: Major:
1. This story is incomplete and is highly descriptive. The importance of how quickly HSVs diversify upon serial passage in vitro is unclear.

We have performed a comprehensive comparative study with an unprecedent level of methodological controls, both experimental- and bioinformatics-wise. All these proceedings have proved to be critical in the assessment of differential generation of genetic variability between these two highly related human herpesviruses.
Bringing to the field a methodological in vitro approach to better understand and analyze how human herpesviruses evolve and adapt to different stimuli, is of high interest and relevance, particularly when there is an ongoing discussion in the field to understand whether this high genetic diversity observed in vivo and in vitro is in fact real of just a technical or methodological artefact.
The fact that we have shown evidence of how differentially HSVs mutate upon serial passage in cell culture, with this unprecedent level of controlled detail and characterization, is of high importance itself, as other authors have pointed out in the herpesviruses field [42, 44, 59] (https://pubmed.ncbi.nlm.nih.gov/31614759/).

2. HSV-1 strain SC16 and HSV-2 strain 333 were chosen. What is the rationale? Would other strains commonly used among researchers display similar genomic diversity over serial passages?

As mentioned in the discussion (lines 505-507), HSV-1 strain SC16 and HSV-2 strain 333 were chosen for their relevance as highly neurovirulent laboratory strains used worldwide. We also chose them because the complete reference genomes used for the variant analyses were generated precisely from the same original stocks used in this study. These reference genomes are complete, and represent with accuracy the structural and genetic heterogenicity contained into each original stock, being suitable for accurate and precise variant analysis. This is mentioned in the discussion (lines 566-574).

We also mentioned in the discussion (lines 535-543) that the lower genetic diversity shown by HSV-1 (compared to HSV-2) may be strain-independent, since in vitro evolution studies using HSV-1 strain F [44] showed similar numbers of de novo mutations to what we found for HSV-1 strain SC16. There are not similar in vitro evolution studies using HSV-2. We would anticipate that different strains of HSV-2 could behave similarly to strain 333, generating higher genetic variability than HSV-1. Further investigation is needed in this regard.

3. It is unclear if the diversity observed is simply a tissue culture phenomenon. While this might prove important and change the way in which researchers handle the virus (in other words, avoid serial passaging on Veros), it is unclear how this actually impacts viral fitness. The authors show one set of images from one clone to describe the differences in plaque formation, but how consistent is this? Is this due to the passage of the Vero cells themselves? Is this relevant to actual changes and genetic diversity in an individual? If this diversity happens much more slowly in individuals (presumably it does), then what is our take away from this tissue culture experiment beyond we shouldn't be passaging virus ad nauseum?

Both HSV-1 clones in both cell types showed lower number of MVs than HSV-2 counterparts, suggesting that this differential generation of genetic diversity is specific for each HSV subtype, rather than an in vitro phenomenon. If the later was the case, it would be observable in both HSV subtypes in both cell types used. This also evidence that rather than a cell type-linked phenomenon (e.g. Vero cell-specific), the differential rate of genetic variability generation is due to each HSV's biological features. Nonetheless, the fact that both HSV-2 clones exhibited a higher number of MVs in HaCaT than in Vero cells, suggests that the different selective pressures due to each cell type physiology may play a role in the amount and speed of genetic variability generation.

Kuny et al. [44] reported that a heterogeneous population of HSV-1 increased its genetic diversity and changed dramatically its phenotype after ten passages in Vero cells, while a plaque-purified population did not. We have reported here that even homogeneous, plaque-purified population of HSV-1 (set of images in Fig 1B) can drastically change its viral phenotype in ten passages, showing that the ability of HSV to mutate is an intrinsic biological feature of its biology (lines 231-234). These images are shown in order to exemplify how just one de novo mutation can change the viral phenotype in a few passages in cell culture. These mutations in UL27, as well as others in UL13, have been reported to favor a syncytial plaque phenotype in Vero cell culture [44]. These evidences of how the in vitro generation of genetic variability consistently impacts HSV viral fitness have been reported and discussed by other authors [42, 59].

We mentioned above, as well as in the introduction (lines 148-155), that it has been reported in numerous studies of how these high levels of genetic variability have also been observed in clinical isolates of HCMV [35-40], HSV-1 [32, 41] and HSV-2 [33]. Even though there is controversy (mostly for HCMV) on whether this genetic diversity is higher in coinfections with multiple distinct strains, the majority of reported studies of HSV suggest that herpesviruses can generate genetic diversity in a few rounds of replication after reactivation from latency.

As commented in the discussion (lines 550 - 560), our in vitro results keep consistency with previously described levels of in vivo genetic diversity between HSV-1 and -2. Ten HSV-1 clinical isolates from Finland exhibited less than 150 grouped MVs [32], while ten neonatal isolates of HSV-2 showed 1821 groped MVs. Most likely, the generation of this in vivo genetic diversity may occur slower than in vitro, due to the host (and niche-wise) selective pressures that the virus must face during a natural infection.

If just one non-synonymous SNP can completely change the viral phenotype, and herpesviruses can adapt to new selective pressures quicker than thought, viral stocks should be prepared and characterized very carefully prior experimental use [42, 59]. More importantly, this may be relevant to prevent resistance to anti-viral drugs (e.g. it was reported that HSV-2 isolates generated drug-resistant mutants 30 times faster than HSV-1 clinical isolates [27]), as well as to better understand how each HSV subtype differentially evolves.

4. The overall effect on viral fitness, growth, pathogenesis is not clear. There is only one experiment that assesses viral growth (at high and low MOI), yet there are no error bars. There is a single experiment in mice, using only 5 animals per infection condition. These data also reveal no statistically significant phenotype (at least none are indicated on the presented graphs). Nonetheless, this work-up is minimal, and does not provide a cohesive picture of the impact of these mutations on viral fitness, growth, or replication.

In this study we have thoroughly characterized how HSV differentially evolve in cell culture, under normalized and controlled conditions. Our experimental design was intended to address the previous question, not to assess how the de novo generated genetic diversity may impact the viral fitness in vivo. The fact that just one non-synonymous SNP can dramatically change the in vitro viral phenotype (shown here and [44]), makes studying the latter challenging. Further studies are needed to fully understand and characterized the overall (MVs profile) but also individualized (specific MVs) effect on each aspect of the virus biology.

We performed replication kinetics and pathogenesis studies of plaque-purified viral clones to corroborate that their biological features were comparable to their original stocks after the reduction of genetic diversity (S4 Fig). Replication kinetics data (S4 Fig A) show means and SD from two independent experiments performed in triplicate. We have included this in S4 Fig legend. It must be noticed that the genetic variably of these clones was reduced by plaque isolation, not increased by passage in cell culture. We specified in the results section (lines 212-220) that the purpose of these studies was to ensure that the MVs detected in each purified clone did not affect its viral fitness significantly compared to their original stocks, which we did not observe. That was a critical step to establish a confident base line prior to evaluate the generation of genetic diversity in cell culture.

Reviewer #3: Overall, the study is robust with significant care taken to address many of the obvious concerns associated with minority variant detection. However, there are several details missing from the Methods section that I would like to see addressed along with one additional analysis.

1. One potential issue with ultra-deep sequencing is that the proportion of PCR duplicated fragments that are sequenced increases. It is therefore important to assess the level of duplicate reads and remove these prior to analysis (e.g. using picard MarkDuplicate). It is not clear to me from the methods whether the authors did this.

Please, see the combined answer in point 3.

2. HSV-1 and HSV-2 genome sequences contain numerous homopolymers that are very challenging for short-read sequencing approaches to deal with as they are prone to PCR errors (during library preparation), and alignment errors. Local realignment strategies can help mitigate the latter and should be implemented.

Please, see the combined answer in point 3.

3. VarScan2, while useful in many respects, relies on input data formatted using the SAMtools mpileup command. There are several issues with the generation of mpileup files, particularly when dealing with small genomes with high depths of coverage. In my experience, this leads to a vast overinflation of MVs called. I think it is thus critical that the authors perform a secondary analysis using an alternative software approach that does not rely on mpileup files. At minimum I would suggest using either LoFreq (https://csb5.github.io/lofreq/) or bamreadcount (https://github.com/genome/bam-readcount). Both are powerful, work directly from BAM files, and in the case of bamreadcount, allow for additional (custom scripted) filtering of variant calls by determining the average position of a SNV/indel within each read (e.g. one can filter out those with a strong 5' or 3' bias) and/or comparing average base call qualities for the parent and variant allele. My expectation is that once these additional analyses are dealt with is that the overall results will not significantly change (i.e. HSV-2 will show greater rates of MV accumulation than HSV-1) but that the numbers of MVs that are robustly detected will be less for most/all samples. If this is not the case and the original results remain consistent then I that is also important to note (i.e. multiple distinct analysis methods generate very similar results) as it lends added weight to the conclusions.

We have performed three new variant analyses using:

- *Picard tools (addressing point 1), GATK (addressing point 2) and VarScan2*
- *LoFreq (addressing point 3)*
- *BAMreadCount (addressing point 3)*

From these new analyses, we have included a comparative table with the total number of MVs detected in each sequenced sample (S15 Table), as well as the de novo MVs (S16 Table). We have also plotted and included these results in a new supplementary figure (S10 Fig). Total number and de novo MVs count did not change dramatically when implementing Picard tools (removing duplicates) and GATK (local realignment) prior to VarScan2 analysis. More importantly, de novo MVs detected with or without this optimization steps were practically identical across samples from the in vitro evolution experiments.

We found a general reduction in the total number of MVs detected by LoFreq and BAMreadCount, with some punctual samples increasing their counts. However, further analysis to detect de novo MVs revealed a consistent pattern compared to VarScan2 results. HSV-2 clones consistently registered greater rates of de novo MVs than HSV-1 counterparts, in every variant caller's analysis. The fact that we observed similar and consistent results across different software's' analysis has added and extra degree of robustness to the confidence in our findings. Results (lines 396-400) and discussion (lines 606-614) have been updated accordingly.

Part III – Minor Issues: Editorial and Data Presentation Modifications

Please use this section for editorial suggestions as well as relatively minor modifications of existing data that would enhance clarity.

Reviewer #1: There are a few areas where the wording in the results is slightly confusing. Examples include line 238 (inexistent at P0 and P5, should read non-existent)

Line 245 .....even low genetically diverse purified viral populations. - Maybe should read populations with low diversity.....

We have corrected the mentioned points.

Reviewer #2: Minor:

1. There are some typos/phrasing that should be corrected by editorial review.

2. Line 92: HSV-1 doesn't always eventually lead to encephalitis. Should rephrase to "and sometimes leads to encephalitis".

We have replaced "eventually" by "occasionally".

3. In the Introduction, the authors state that diversity can be generated over multiple cycles of latency and reactivation (lines 145-146). This is certainly possible even in the non-genital context. However, I don't think this can be connected to passaging of stocks in tissue culture (as implied on the following line).

As mentioned in line 167, Kuny et al. [44] have shown evidence of how a heterogeneous population of HSV-1 can increase its genetic diversity and change its phenotype after ten passages in Vero cells.
We observed the de novo appearance and raise of a similar syncytia-forming variant in one clone of HSV-1, among other de novo generated MVs. Together with the data presented in this study, we think there are consistent and supportive evidence to believe that HSV can generate genetic diversity, which may alter its viral phenotype, after sequential passage in tissue culture.

4. At first mention of HaCaT (line 181), add a brief description of what these cells are, so readers don't have to dig in the Methods.

We have specified that HaCaT cells are human keratinocytes (line 188).

Reviewer #3: 1. The final statement presented in the discussion and abstract suggests that differences in the rate of MV generation in HSV-1 and HSV-2 may be due to evolutionary divergence associated with adapting to difference anatomical niches. I am not sure the evidence supports this line of speculation over other possibilities, particularly in lieu of previous studies into the origins of HSV-1 and HSV-2 e.g. https://www.ncbi.nlm.nih.gov/pmc/articles/PMC4137711/ - which should also be cited/discussed in the introduction at least.

Although we agree that our data do not demonstrate that replication in different anatomical niches may account for the differences observed between HSV-1 and HSV-2, we think that bringing some speculative hypotheses is appropriate to drive future research. As suggested by the reviewer, we have discussed and cited this article in the introduction and discussion, speculating that the preexisting differences between HSV-1 and HSV-2 due to a hypothetically distant evolutionary origin, together with the differential selective pressures of each anatomical area, might have contributed to a higher evolutionary divergence, which could be exemplified as differences in the rate of MV generation.

2. Introduction – lines 138 – 150. The authors are conflating the generation of genetic diversity with evolutionary rate and also cite several papers suggesting that herpesviruses have highly diverse populations in vivo. 'Highly' is doing a lot of heavy lifting in this statement and the authors fail to cite any papers that counter this argument (e.g. https://www.pnas.org/content/116/12/5693), nor do they discuss the informatics challenges presented by short-read sequencing approaches, particular in regard to robust and reliable variant calling (a significant problem). More balance is thus required in this part of the text.

The mentioned paper described that the high human cytomegalovirus intrahost diversity found in clinical isolates may be caused by coinfection with multiple distinct strains [39], while other authors argued that this diversity was similar in mixed and single infections [37].
To the best of our knowledge, this matter has not been properly address for HSV-1 and HSV-2, where some studies have scratched the surface of it by reporting the first evidence of a single individual shedding two distinct HSV-2 strains with discrete but detectable variability

(https://pubmed.ncbi.nlm.nih.gov/29281620/); and demonstrating changes in the HSV-2 genome over time in a recently infected host [61].

Nevertheless, we have changed "highly" by "dynamic" (line 145), and included/cited these human cytomegalovirus studies to show that there is controversy in this regard in the field (lines 148-152). We have also briefly commented about the bioinformatic challenges presented by short-read sequencing of high G + C content repetitive genomes (lines 616-617).

3. Fig. 2 - the authors present an analysis of synonymous and non-synonymous SNV (panels A & C) but this is problematic given that the full coding diversity of HSV-1 (and likely HSV-2) is far more complex than can be ascertained from the existing GenBank annotation (https://www.nature.com/articles/s41467-020-15992-5). This is also problematic when defining coding versus non-coding regions.

In Fig 2 (as well as in Fig 4 and S2 Table), we have included total MVs detected, categorizing them by coding and non-coding regions. By doing this, we have accounted for all the genetic variability found in each sample but not only in that localized in coding regions (synonymous and non-synonymous SNV). We understand that this takes into consideration the fact that the current genome annotation does not represent the full coding capacity of HSV-1, but it does represent its full diversity in terms of MVs detected.

4. Fig. 5 - the authors refer to 'genes' when in fact I think they mean open reading frames (ORFs).

The canonical predicted 80 open reading frames (ORFs) of HSV are usually referred as viral genes in the literature [23, 32, 33, 44, 45].
